# Immune-endocrine crossroads: the impact of nuclear receptors in Tuberculosis and Chagas disease

**DOI:** 10.3389/fendo.2025.1538376

**Published:** 2025-02-07

**Authors:** Ana R. Pérez, Oscar A. Bottasso, Natalia E. Santucci

**Affiliations:** ^1^ Laboratorio de Estudios en Enfermedad de Chagas, Instituto de Inmunología Clínica y Experimental de Rosario (IDICER)-Consejo Nacional de Investigaciones Científicas y Técnicas (CONICET)-Universidad Nacional de Rosario (UNR), Rosario, Argentina; ^2^ Facultad de Ciencias Médicas, Universidad Nacional de Rosario (UNR), Rosario, Argentina; ^3^ Laboratorio de Estudios en Tuberculosis, Instituto de Inmunología Clínica y Experimental de Rosario (IDICER)-Consejo Nacional de Investigaciones Científicas y Técnicas (CONICET)-Universidad Nacional de Rosario (UNR), Rosario, Argentina

**Keywords:** NR, GR, RAR/RXR, PPAR, LXR, VDR, tuberculosis, Chagas disease

## Abstract

Nuclear Receptors (NRs) comprise a superfamily of proteins with essential roles in cell signaling, survival, proliferation, and metabolism. They act as transcription factors and are subclassified into families based on their ligands, DNA-binding sequences, tissue specificity, and functions. Evidence indicates that in infectious diseases, cancer, and autoimmunity, NRs modulate immune and endocrine responses, altering the transcriptional profile of cells and organs and influencing disease progression. Chronic infectious diseases, characterized by pathogen persistence, are particularly notable for an exaggerated inflammatory process. Unlike acute inflammation, which helps the host respond to pathogens, chronic inflammation leads to metabolic disorders and a dysregulated neuro-immuno-endocrine response. Over time, disturbances in cytokine, hormone, and other compound production foster an unbalanced, detrimental defensive response. This complexity underscores the significant role of ligand-dependent NRs. Tuberculosis and Chagas Disease are two critical chronic infections. The causative agents, *Mycobacterium tuberculosis* and *Trypanosoma cruzi*, have developed evasion strategies to establish chronic infections. Their clinical manifestations are associated with disrupted immuno-endocrine responses, pointing to a potential involvement of NRs. This review explores the current understanding of NRs in regulating immune-endocrine interactions within the context Tuberculosis and Chagas Disease. These diseases remain significant global health concerns, particularly in developing countries, highlighting the importance of understanding the molecular mechanisms underlying host-pathogen interactions mediated by NRs.

## 1 Introduction

Nuclear Receptors (NRs) were identified as ligand-dependent transcription factors that respond to hormones and other metabolic ligands, displaying genomic and non-genomic activities. These receptors have evolved in an intricate network of multiple molecular pathways, participating in physiological processes such as metabolism, reproduction, development, and immune response. Since homeostasis depends on integrated physiology based on communication between organs, tissues, and cells, NRs are likely to play a major role in this scenario. As such, NRs gained great interest in the field of biomedical sciences and drug discovery, as they are also involved in the pathophysiology of several diseases (1).

The NRs superfamily is classified into six subfamilies based on their evolutionary sequence conservation. The larger group includes thyroid hormone receptor-like members such as the Vitamin D Receptor (VDR), Thyroid Receptors (TR), Peroxisome Proliferator-Activated Receptors (PPAR), and Liver X Receptors (LXR), while Retinoid A Receptor (RAR), Retinoid X Receptor (RXR) members and HNF-4 constitute a different subfamily. Another group comprises sex and adrenal Steroids Receptor-like members, including Glucocorticoid Receptor (GR), Mineralocorticoid Receptor (MR), Progesterone Receptor (PR), Androgen Receptor (AR), Estrogen Receptor (ER) and Estrogen-related receptors (ERR). The fourth group consists of Nerve Growth Factor I-B (NGFI-B)-like group members, including NGFI-B, NUR77, NURR1, and NOR1 that belong to the NRS4A subfamily. The fifth group includes Steroidogenic factor-like receptors, NRS5A1 and NRS5A2. And, finally, there is a subfamily that contains a single type of receptor unfitting for the previous group´s criteria, the Germ Cell nuclear factor-like NRS6A1 or GCNF1 (2, 3). An important issue is that some NRs are classified as “orphan” due to the lack of identified natural ligands. Among these, the well-studied ones include the ERRs, NRS3B1, 2, and 3, and the Steroidogenic Factor 1 (SF1) (4). When their cognate ligands are identified, orphan NRs become “adopted” and are referred to as adopted NRs. Those NRs whose ligands were identified were called “classic” NRs, and generally are endocrine receptors like Thyroid Hormone Receptors or Estrogen Receptors (5).

The NRS structure is subdivided into four functional areas. The N-terminal part (domain A/B) is the most variable in size and protein sequence. It comprises the activating function-1 (AF-1) domain, responsible for the interaction with co-activators and co-repressors (regardless of the ligand presence), whose intervention is essential for the transcriptional activity of the receptors (4). The central area called the C domain, is the most preserved regarding the NRs superfamily and contains the DNA-binding site (DBD). NRs modulate transcription of their target genes binding to specific promoter sequences in the form of homodimers, heterodimers, or monomers (4). Another important region is the D domain, a flexible hinge region connecting the DBD to the ligand-binding domain (LBD) that allows the rotation of the LBD to facilitate attachment of the dimer on direct or indirect type responses. It also contains a nuclear localization signal (NLS) which facilitates the receptor´s transfer to the nucleus (4). Finally, the E domain contains an LBD which confers the specificity and selectivity of the physiological response. At the carboxy-terminal end of the LBD, there is a second transcription activation domain (AF-2) that, after ligand binding, goes through a conformational change that allosterically controls the interactions between receptor and coactivators and corepressors (4, 6, 7).

Considering NR´s ligands, unlike most intercellular molecules, they can cross the plasma membrane because their lipophilic nature and interact with their cognate receptors inside the cell. There is a group of NRs, endocrine NRs, with a high affinity for hormones which, in the absence of ligand, are usually cytoplasmic and monomeric. They are bound to heat-shock protein chaperones that, after ligand binding, dissociate from them, homodimerize, and translocate into the nucleus, activating then the transcription of their respective target genes. On the other hand, orphan NRs and adopted orphan NRs (with “adopted orphan” meaning that the physiological ligand/s were identified for an “orphan” receptor), generally have lower affinity for fatty acid or phospholipid-based ligands. It is unclear whether all these receptors have *bona fide* ligands, considering that some NRs can also act in their absence. Orphan receptors are often constitutively nuclear and used to form heterodimers with RXR. The common thread throughout NRs regulation is their ability to bind a hydrophobic, lipid-based ligand, resulting in an altered conformation of the LBD which, in turn, alters the coactivator recruitment and the localization of the receptor, changing the transcription programme (7, 8). The functions of NRs can also be modulated by post-translational modifications that include phosphorylation, ubiquitylation, acetylation, methylation, and SUMOylation. These modifications can regulate receptor protein stability, intracellular location, and DNA-binding properties allowing the crosstalk between NRs and cell surface receptor signaling pathways (9).

The importance of NRs in physiology and pathophysiology makes them suitable candidates as therapeutic targets for several diseases, such as hormone-dependent cancers, and type 2 diabetes mellitus, as well as those with an inflammatory, autoimmune, or malignant basis. Research on them certainly will allow the designing of diagnostic and prognostic tools for understanding the aetiology and the progression of several diseases (1, 9). As they bind to small molecules, they represent a promising therapeutic target for which selective agonists and antagonists can be engineered. The significance of NRS-regulated pathways in sustaining a physiologic balance is highlighted by the fact that over 10% of US Food and Drug Administration (FDA)-approved drugs are targeting one of the 48 known human NRs (10). Therefore, further research is needed to identify the potential long-term secondary impacts of modulating these receptors during extended treatment.

This review explores the current understanding of NRs in regulating the immune-endocrine and metabolic response, focusing on two chronic infectious diseases -Tuberculosis (TB) and Chagas Disease (CD)- which remain significant health challenges, particularly in developing countries.

## 2 Nuclear receptors in health and disease

A key aspect of the regulation exerted by NRs lies in their integrative roles in development and homeostasis, including their ability to regulate diverse genes across various cell types, thereby supporting the specialized functions of different organs. The presence of a pathological stimulus is known to result in increased transcriptional activity, mostly associated with transcription factor activities attempting to eliminate the harmful agent and restore homeostasis. In this scenario, NRs play a major role in both physiology and pathophysiology. Products of stromal and immune cells shape the function of the immune system through cell-surface receptors and NRs (1, 7, 11).

NRs are mostly regulated endogenously by several small lipophilic ligands such as steroids, retinoids, phospholipids, oxysterols, and vitamins, whose union induces conformational changes, with these receptors binding at specific DNA sequences throughout the genome (10). After the interaction, co-regulators, diverse proteins that remodel the chromatin and the general transcriptional machinery are recruited to activate or repress target gene expression. In this regard, NRs are responsible for the strict regulation of thousands of genes, and their aberrant expression or activity became involved in the causation of several diseases such as cancer, autoimmunity, or chronic inflammation (11).

In the context of infectious diseases, particularly chronic ones, it is important to examine the role of specific NRs in host-pathogen interactions and their subsequent impact on the immune-endocrine and metabolic responses. In a broad sense, the host’s response to infectious agents involves the generation of an inflammatory response addressed to eliminate the pathogen, promoting tissue repair and restoring functionality and tissue homeostasis. This response is accompanied by endocrine and metabolic adjustments. In this regard, immune cells exhibit diverse subpopulation profiles through transcriptional reprogramming, partly regulated by NRs (10, 12). Numerous NRs have been extensively studied regarding their participation in the polarization and function of myeloid lineage cells, with some of them displaying a specific profile (13).

Next, the role of NRs in neuro-immune-endocrine regulation will be examined, followed by a discussion of their involvement in TB and CD (14–18). Since bioinformatics and molecular biology studies demonstrate that chronic infections show significant enrichment for transcription factors, including NRs (19), we will focus on NRs like GR, PPARs, RXR, RAR, LXR, and VDR. This selection is based on the abundance of existing research on these NRs. Although the study of NRs is an evolving field with emerging findings on their roles in various pathologies, we concentrate on receptors with significant evidence of involvement in immunoendocrine regulation during chronic infections.

### 2.1 GR receptor (NRS3C)

GR is, by far, one of the most studied NRs. This endocrine receptor binds glucocorticoids (GCs), which are essential steroid hormones for the daily functioning of mammals. They are involved in several physiological processes, namely in metabolism, immune response, growth, cardiovascular function, mood and cognitive functions, reproduction, and development (14). GCs along with aldosterone (a mineralocorticoid hormone) and dehydroepiandrosterone (DHEA) are synthesized in the cortex of the adrenal gland, from a common precursor, cholesterol (14). This receptor is mainly cytoplasmic and is associated with a chaperone complex. Upon GCs binding, the GR is released from the chaperone complex and translocated into the nucleus, where the receptor has two major genomic actions. GR activates target genes containing GC response elements (GREs) in their regulatory regions, binding as a homodimer. This mechanism promotes cell-type-specific expression of anti-inflammatory and pro-apoptotic genes and also genes involved in gluconeogenesis or lipogenesis, leading to metabolic side effects. Conversely, GR can inhibit the expression of pro-inflammatory or pro-survival transcription factors such as NFκB, AP-1, and STAT (15).

After infection, a strong pro-inflammatory response normally occurs characterized by the release of TNF-α, IL-1β and IL-6 by activated immune cells, a characteristic of diseases such as TB and CD (17, 18, 20–22). These cytokines gain access through the circulation to the central nervous system triggering the Hypothalamic-pituitary-adrenal (HPA) axis activation, with the concomitant secretion of Corticotropic Releasing Hormone (CRH), followed by the production of Adrenocorticotropic hormone (ACTH) and then, the stimulation of GCs synthesis by the adrenal gland. GCs exert both inhibitory and stimulatory effects on many issues of the immune response (23–25). As stated, GCs affect the expression of genes for proinflammatory cytokines through various mechanisms, i.e., inhibition of Nuclear Factor kappa B (NF-κB) signaling, and modulation of the activity of Th1 and Th2 cells (26, 27).

The more relevant isoforms of GR in immunomodulation are GRα and GRβ. GCs exert their biological effects upon interacting with GR, which promotes the translocation of these receptors to the nucleus and binding to GREs sequences, or with negative GREs (nGREs) which up or down-regulate gene expression, respectively. Also, GRs interact with other transcription factors or both possibilities. The GRα dimer mediates immunological cortisol functions, although the GRα/GRβ hetero dimer acts as a negative dominant since GRβ does not bind to a ligand altering GRα functioning (28–30).

### 2.2 Retinoids receptors (NRS1B/RAR AND NRS2B/RXR)

Retinoids, which include natural vitamin A and its synthetic derivatives, have a wide spectrum of biological actions and are required for the optimal functioning of the immune system. So far, two families of nuclear retinoid receptors have been described, the Retinoid X Receptor (RXR) and the Retinoid Acid Receptor (RAR), sharing only 29% homology in their ligand binding domains (31). Diversity in gene expression control by Retinoid signals arises from the complexity within the signaling pathway. A key source of this diversity is the presence of two families of RARs/RAR isotypes (alpha, beta, gamma) and RXR isotypes (alpha, beta, gamma), along with their numerous isoforms that form RXR/RAR heterodimers binding to variable cis-acting response elements of Retinoid Acid target genes. Additionally, cross-modulation with cell-surface receptor signaling pathways and the interaction of RARs and RXRs with various coactivators and corepressors contribute to the complexity and combinatorial effects that underlie the pleiotropic actions of retinoids (31).

RXR plays a pivotal role within the NRs superfamily, and all isotypes selectively bind the Vitamin A derivative 9-cis Retinoid Acid. Each RXR isotype exists in multiple isoforms with distinct tissue distributions and developmental expression patterns (32). These receptors are a particular subclass of NRs known for their unique dimerization properties. They form heterodimers with about one-third of known human NRS, most of which require RXR as an essential partner for DNA binding and transcriptional regulation (i.e., TR, PPAR, RAR, VDR, LXR) (10, 33). In this regard, RXRs influence various genetic programs, including cell differentiation, immune response, and lipid and glucose metabolism. Their versatility arises from forming heterodimers classified as permissive or nonpermissive groups. In the first case, ligands of either partner can activate them, allowing RXR agonists to induce transcriptional change in multiple NRs pathways simultaneously. Conversely, nonpermissive heterodimers can only be activated by agonists of the dominant partner receptor, not by RXR agonists (10, 34). For instance, the PPAR/RXR heterodimer is a key mediator of 9-*cis*- Retinoid Acid (9CRA) action and signaling by fatty acids and their derivatives (32, 35).

RXR heterodimers normally bind to target genes without ligand, mediating transcriptional repression by recruiting corepressor complexes via interactions with NRS corepressor proteins. Some RXR partners can also repress transcription in a ligand-dependent manner, explained by two mechanisms. First, certain corepressors bind to nuclear receptors in an agonist-dependent manner, inhibiting target gene expression. Second, negative regulation can occur by inhibiting the activity of other signal-induced transcription factors, such as NF-kB or AP-1. This ligand-dependent transrepression highlights an intriguing interplay between different signaling pathways (10).

Regarding RARs, the three subtypes form obligate heterodimeric complexes with the three RXRs to respond to their specific ligands and exert their pleiotropic functions. The heterodimers act as ligand-dependent transcriptional regulators by binding to the specific Retinoic Acid Response Element (RARE)sequences found in the promoter region of retinoid target genes (36, 37). In the absence of RAR agonists, the RXR/RAR heterodimer recruits a corepressor complex made up of proteins like NCoR or SMRT, along with factors such as histone deacetylases or DNA methyltransferases, inhibiting transcription. RAR agonists bind to induce conformational changes that recruit coactivator complexes and epigenetic factors while releasing corepressors. They can autonomously activate transcription whereas RXRs cannot respond to its agonists when RAR ligands are absent, showing a clear subordination of RXR to RAR. However, there is a synergistic transcriptional activation when both partners are simultaneously bound to agonists, revealing that RXRs are not transcriptionally silent partners (37).

Natural retinoids are synthesized from the oxidation of Vitamin A. All-trans Retinoic Acid (ATRA) exhibits a high affinity for RARs and is the most biologically active retinoid in mammals. In addition to ATRA, 9CRA, an isomer of ATRA, also acts as a ligand for RARs. While ATRA exclusively binds to RAR, 9CRA can bind to RAR and RXR (10, 37). These molecules seem to have anti-inflammatory and tolerogenic effects, together with some tissue-restricted mechanisms promoting an adaptive IR. Vitamin A has been shown to foster the expression of gut-homing receptors on activated T and B cells and the production of Immunoglobulin A in a tissue-restricted mechanism. Besides, RA enhances the induction of T regulatory cells FoxP3^+^ exerted by Transforming Grow Factor β (TGF-β) while suppressing the differentiation of Th17 cells (38, 39). Some studies demonstrated that ATRA modulates innate immunity with a central function in the differentiation and migration of Dendritic Cells (DC). And, in a proinflammatory context, ATRA also influences the Ag-presenting capacity of DCs. The positive or negative influence of ATRA seems to be dependent on the proinflammatory context and/or the type of DC (40). Indeed, these cells in the presence of IL-4 and GM-CSF (Granulocyte Macrophage-Colony Stimulation Factor) produce RA, in autocrine and paracrine signaling (39, 40). It is worth noting that, despite tolerogenic and anti-inflammatory effects, the influence of Retinoic Acid on effector T cells depends on the microenvironment, which might act as an adjuvant to produce certain cytokines. During infection or tissue damage, Retinoic Acid can induce a proinflammatory phenotype in DCs, releasing IL-15, IL-12 and IL-23 (41).

Regarding Mθs, Retinoids appear to have an anti-inflammatory effect, with suppressive effects on inflammatory disease models. ATRA treatment suppressed TNF-α, IL-12, and nitric oxide production of activated Mθs and increased production of the immunoregulatory cytokine IL-10, presumably polarizing Mθs toward an anti-inflammatory phenotype (10). Although the molecular mechanisms involved are not completely identified, RXR activation by 9-cis-RA decreased IL-12 production. On the other hand, Mθ lacking RXRα shows reduced levels of CCL6 and CCL9, impairing leukocytes recruitment to sites of inflammation (10).

### 2.3 PPAR receptors (NRS1C sub-family)

In 1990, Issemann and Green (42) identified a new member of the steroid/thyroid/vitamin superfamily of nuclear receptors in mouse liver, which they named Peroxisome-Proliferator Activator Receptor (PPAR) due to its activation by various peroxisome proliferators. PPAR receptors are a well-studied family of fatty acid-activated NRs consisting of three members: PPARα, PPARγ, and PPARδ (also designated as PPARβ). They are known by their participation in fatty-acid metabolism and adipocyte differentiation, since interact with various non-esterified and polyunsaturated fatty acids, prostanoids, or eicosanoids, converting these lipid signals into transcriptional programs that regulate multiple aspects of lipid metabolism, including synthesis, transport, storage, mobilization, and oxidation (10).

Each member displays distinct ligand preferences due to variations in their binding pockets’ size or lipophilicity. Natural ligands for PPARs include lipid-derived metabolites, such as dietary lipids that can activate them (43). As with many NRs, these receptors bind to their corresponding Response Element (PPAR Response Element) as an obligate heterodimer with RXR (44). Regarding their expression, PPARα is predominantly expressed in the liver, PPARγ in adipose tissue, macrophages, and dendritic cells, whereas PPARβ/δ is ubiquitous. PPARα and PPARβ/δ are highly expressed in oxidative tissues and regulate genes related to substrate delivery, oxidation, and oxidative phosphorylation. In contrast, PPARγ primarily promotes energy storage by enhancing adipogenesis and lipid synthesis, with the highest expression levels found in white adipose tissue and immune cells (45).

PPARγ is the most extensively studied because of its importance as a regulator of adipose tissue development, fatty acid synthesis, insulin sensitivity of major glucose-utilizing tissue, and its immunomodulatory role in Mθs and DCs functions. In Mθs, PPARγ regulates polarization, maturation, epigenetics, and metabolism, whereas in DCs is a central regulator of functional maturation, particularly in the immune tolerance, being also critical for the regulation of adaptive immune cells, to damp excessive inflammatory response (5, 43, 46). It is widely accepted that the potential mechanism responsible for this anti-inflammatory effect is trans-repression, by which PPARγ interacts with transcription factors involved in pro-inflammatory signaling pathways, such as AP-1 or NF-kB, leading to a failed induction of inflammatory response mediated by these transcription factors (10). Conversely, under inflammatory conditions, Mθs tends to down-regulate PPARγ. However, PPARγ is required during the resolution phase of the inflammatory response, and loss of PPARγ is associated with sustained immune response (13).

PPARγ, although not essential for monocyte/Mθ differentiation, functions as an important modulator of their lipid metabolism and immune functions. It is known that agonists of this receptor inhibit proinflammatory cytokine production affecting the expression of cytokines, such as TNF-α, IL-1β, IL-6, and IL-12, and of enzymes mediating bacterial killing and tissue damage, such as inducible nitric oxide synthase (iNOS) and MMP-9, respectively. Besides, PPAR γ inhibits the expression of monocyte chemotactic protein 1 (MCP-1) and its receptor CCR2 in Mθs which might help to retain these cells in sites of inflammation (5). Concerning the role of PPARγ in DC differentiation, the participation of the receptor is controversial. On one hand, the pharmacological blockade of PPARγ in human monocytes turns GM-CSF into a potent inducer of DC differentiation (47). On the other hand, Matsuba et al. demonstrated an up-regulation of PPARγ and its associated genes when DCs are obtained from mouse bone marrow culture (48).

Concerning PPARα, it appears to potentiate the polarization of macrophages toward an anti-inflammatory phenotype, as PPAR γ does. Regarding T cell response, PPARα plays an important role in the development of T cell-mediated autoimmune diseases, in a gender-specific manner. The effects of PPARβ/δ on Mθs are less well established, with a certain background dependence. Considering T cell development, it is well established the role of PPARδ in inducing tolerance and preventing autoimmunity (43). Since PPARs are implicated in a variety of human diseases such as cancer, and metabolic and autoimmune conditions, the therapeutic targeting of them with synthetic exogenous ligands has been attempted in several of these disorders. In fact, the employment of PPARα ligands resulted in less inflammation-related symptoms and disease severity in several models, including allergic airway disease, arthritis, non-alcoholic fatty liver disease, type 2 diabetes mellitus, and inflammatory bowel disease (49–53).

### 2.4 LXR receptors (NRS1H subfamily)

The LXRs were initially described in the liver, as their name suggests. However, in the last years it was established the importance of these LXRs in the interconnection between metabolism and the immune system. The LXRα and LXRβ function as a critical signaling node linking lipid metabolism, inflammation, and immune cell function. LXRs, like PPARs, bind to DNA as heterodimers with RXRs. LXRα is the dominant subtype and is highly expressed in the liver and in tissues that play roles in cholesterol metabolism, including the intestine, adipose tissue, kidney, and adrenals, whereas LXRβ is ubiquitously expressed. Regarding the immune system, LXRα is restricted to the myeloid lineage while LXRβ can be found in all cell types (54). Once activated, LXRs induce the expression of an array of genes involved in cholesterol absorption, efflux, transport, and excretion. In addition to their function in lipid metabolism, LXRs have also been found to modulate immune and inflammatory responses in Mθs (55). They are involved not only in the regulation of Mθ cholesterol homeostasis but also in the regulation of their inflammatory response, phagocytosis, and apoptosis. LXR activation prevents cholesterol overload in these cells by simultaneously inhibiting cholesterol uptake and increasing cholesterol efflux (10). The binding of their natural agonists (cholesterol derivatives including oxysterols and cholesterol precursors) induces a conformational change that decreases the affinity of LXR for transcriptional corepressor proteins and increases the affinity for transcriptional coactivators (54).

The identification of LXRs, as important regulators of lipid metabolism, has prompted investigations into the therapeutic potential of the receptors in diseases associated with dyslipidaemia, particularly type 2 diabetes mellitus and cardiovascular disease. In experimental models, LXR agonists both decrease hyperglycemia and improve insulin sensitivity, with inhibition of hepatic glucose production accounting for most of the LXR anti-diabetic activity. Regarding atherosclerotic cardiovascular disease, the recruitment of Mθs to the underlying endothelial layer of blood vessel walls and the uncontrolled uptake and accumulation of oxidized/modified forms of cholesterol by these cells are prominent characteristics of its physiopathology. An inflammatory response would lead to foam cell formation and the initiation of atherosclerosis. The ability of LXRs to promote Mθ cholesterol efflux is of great interest in the therapeutic potential of LXR ligands for the treatment of cardiovascular disease. However, the finding that pharmacological activation of LXRβ alone is sufficient to reduce atherosclerosis while LXRα mediates hyperlipidemic effects has motivated the search for LXRβ-selective agonist (54, 55).

Regarding the immunoregulatory role of LXR, mutual interactions were observed between LXR activation and pathogen-induced inflammation in Mθs. It was demonstrated that, after LPS stimulation, endogenous and synthetic LXR ligands inhibit the expression of inflammation-related genes. This effect can be explained by the observation that a similar transrepression mechanism exists for LXR as for PPARγ. It was reported that ligand binding results in the SUMOylation of the receptor, which inhibits LPS-induced corepressor clearance from the promoter of inflammatory genes. Another piece of evidence is that TLR4, mediating LPS recognition, was identified as a direct LXR target gene, and LXR activation leads to the induction of TLR4 expression in human Mθs (10). Crosstalk between LXR and TLRs may explain how microbial infections disrupt cholesterol metabolism, highlighting LXRs’ role in integrating inflammatory and metabolic signaling (55).

Finally, regarding innate immunity, several studies indicate that, in addition to inducing genes involved in reverse cholesterol transport, LXRs reciprocally repress a set of inflammatory genes such as those involved in the generation of TNFα, iNOS, COX2, IL-6, and IL-1β, the chemokines monocyte chemoattractant protein (MCP)-1, MCP-3, and MMP9. However, the mechanism underlying the repression of inflammatory genes by LXRs is poorly understood. LXR Response Elements have not been identified in the proximal promoters of the repressed genes, suggesting an indirect mechanism. In addition to possible competition for transcriptional coactivators, the body of evidence suggests that inhibition of the NF-κB pathway is involved (55). On the other hand, Gosselet and colleagues demonstrated that, under inflammatory conditions, which may be involved in neuroinflammatory diseases, TNFα triggers the LXR signaling pathway, thus increasing cholesterol efflux. This cytokine also induces 25-hydroxycholesterol production, a cholesterol metabolite mainly produced during inflammatory and infectious conditions, involved in the immune response and intracellular cholesterol metabolism (56, 57). Furthermore, LXR may improve the bacterial killing capacity of macrophages and directly increase TLR4 expression, enhancing macrophage responsiveness to LPS stimulation, and highlighting LXR’s contextual role in innate immunity (58, 59).

### 2.5 Vitamin D receptor (NRS1I1)

Vitamin D has a crucial physiological role in regulating the expression of various genes involved in cellular homeostasis, differentiation, and immune system modulation. The main Vitamin D metabolites are cholecalciferol (VD3) and ergocalciferol (VD2), with VD3 as the predominant bioactive form, naturally synthesized by the skin following exposure to UV radiation. To a lesser extent, an animal-based diet is also a source of this vitamin. As a pro-hormone, Vitamin D should be activated to exert its biological effects, with two hydroxylation steps to metabolize the active form of the vitamin from VD2 and 3 (60). Vitamin D regulates calcium homeostasis and phosphate metabolism through the interaction with their receptor (VDR). It also has a huge impact on the proper functioning of musculoskeletal, immune, nervous, and cardiovascular systems, controlling calcium metabolism, cell growth, differentiation, apoptosis, and adaptive/innate immune responses (60, 61).

Since most human tissues express the VDR gene, the physiological impact of VD results in the regulation of several hundred target genes per VDR expression tissue (62). Vitamin D metabolizing enzymes and VDR are present in many cell types including antigen-presenting cells, T cells, B cells, and monocytes, with different expression levels, implying that Vitamin D could regulate immune response. This vitamin boosts innate immunity against infectious agents and modulates adaptive immune responses through the induction of an anti-inflammatory and tolerogenic microenvironment to limit inflammatory or unwanted immune responses (60). The immunomodulatory effects of Vitamin D include switching between cell-mediated response (Th1) and humoral immunity (Th2), Mθ activation, and production of antimicrobial peptides (63). Regarding VDR, it forms a heterodimer complex with RXR and binds to specific DNA sequences called VDRE (Vitamin D Response Elements) in the promoter region of target genes. In the immune system, Vitamin D exerts its immunomodulatory functions regulating genes involved in immune cell differentiation, maturation, metabolism, and response to cytokines and chemokine (60).

The physiological profile of VDR resembles the endocrine members of the superfamily, such as RAR, GR, and others. Many NRs exhibit a tripartite relationship, as the genes encoding specific metabolic enzymes and transporters that regulate ligand concentrations are targets of these receptors. This indicates a coevolution between metabolic enzymes, transporters, and their regulating NRS, representing a finely tuned system of receptors, ligands, and enzymes that supports genomic signaling for VD and other endocrine hormones and nutritional regulators (62).

## 3 NRS involvement in chronic infectious diseases: Tuberculosis and Chagas disease

Both TB and CD are considered immuno-mediated pathologies (**Box 1**). In addition, both infectious diseases also triggered in parallel to immune reaction a complex neuro-endocrine response against the pathogen, which overall is detrimental to the affected tissues, coupled with the fact of poorly treatment compliance in the case of TB, or the lack of clearly effective drugs for chronic CD (12–16). In this context, key NRs seem to be involved in the pathogenesis of the above-mentioned paradigmatic infectious diseases.

Box 1 **Main clinical and pathological features of tuberculosis and chagas disease.**

→Tuberculosis (TB) is one of the most important infectious diseases worldwide. It is estimated that 2 billion persons are infected with *Mycobacterium tuberculosis*, and 8 to 12 million new cases of active tuberculosis occur each year, accounting for 2-3 million deaths annually. Most people infected with *Mycobacterium tuberculosis* have a clinically latent infection, which remains dormant constituting asymptomatic and non-contagious carriers. The development of clinical post-primary TB occurs in 5%-10% of latently infected persons. Pulmonary disease is the most common form of post-primary TB. When active TB develops, disease localization, severity, and outcome are highly variable but usually present as a pulmonary disease. The clinical spectrum of pulmonary TB ranges from a few foci affecting the upper parts of the lungs to intense tissue destruction and caseous necrosis, which usually disintegrates forming cavitary lesions. Such different disease outcomes are thought to result from complex interactions between *Mycobacterium tuberculosis* and the specific immune response. Resistance to mycobacterial infections is known to be conferred by T cell-mediated immune mechanisms involving cytokines like IFN-γ that ultimately lead to the recruitment and activation of monocyte/macrophage cells possessing an enhanced state of microbicidal activity (64, 65). TB can also affect the endocrine and metabolic response in diverse ways (21).
→Chagas disease (CD) is a parasitic infection caused by the protozoan *Trypanosoma cruzi*, usually transmitted to humans through the bite of a triatomine bug. Currently, it has a worldwide distribution affecting at least 8-10 million people throughout South and Central America, with more than 300,000 cases in USA, and 80,000 in Europe. The major complications of CD are mega syndromes of the gastrointestinal tract and particularly the heart involvement. About 30% of individuals infected with *Trypanosoma cruzi* develop chronic chagasic cardiomyopathy (CCC), resulting in severe heart disorders, which cause approximately 15,000-50,000 deaths annually. The pathogenesis of CCC is still controversial, but the immune response contributes significantly to this pathology. Different mechanisms have been proposed to explain the development of cardiac complaints occurring in chronic CD. The fact that signs of the disease are evident in tissues where parasites are nearly absent gave support for the autoreactive component. Nevertheless, autoimmunity does not entirely explain CCC (66). For instance, parasite persistence, which not only results in chronic inflammatory reactivity but also induces immune responses against parasites and self-tissues as well as the eventual damage accompanying these responses. In addition, endocrine and metabolic alterations are detected in infected individuals, whose importance in the development of the CCC still remains to be evaluated (16).

### 3.1 Tuberculosis

#### 3.1.1 GR

In chronic conditions, like TB, sustained activation of the HPA axis due to ongoing inflammation can lead to desensitisation of GCs in target tissues by down-regulating the GR (67). Former studies from our group in patients with TB showed an increase in GCs plasma levels accompanied by a significantly decreased GRα/GRβ ratio expression in peripheral blood mononuclear cells (PBMCs), mainly in cases with progressive disease (68, 69). Patients with progressive forms of TB, particularly severe cases present a circulating profile characterized by an increased expression of anti-inflammatory-positive-GR-regulated genes (ANXA1 and NF-κB inhibitors) as well as the GRβ isoform and IL-1β, together with a decreased specific proliferative capacity *in vitro*. This scenario suggests that although elevated cortisol levels aim to reduce inflammation, proinflammatory mediators continue to exacerbate the response. Disrupted cortisol levels may also impact circulating cells and the antigen-specific cellular response by impairing mycobacterial-driven lymphoproliferation (70).

#### 3.1.2 RAR/RXR

Vitamin A deficiency has encouraged many studies regarding their implications in various disease states. In the case of TB, there is some evidence pointing out that Vitamin A deficiency is associated with the risk of incident TB (71, 72). However, other studies showed that in human and murine models of TB, supraphysiologic doses of Vitamin A and Retinoic Acid appear to influence *Mycobacterium tuberculosis* growth *in vitro (* 73). It has been observed that the mycobacteria express aldehyde dehydrogenase enzymes, essential in Retinol synthesis, with the possibility of certain metabolization of Vitamin A from the mycobacteria (72). Indeed, a recent study showed that this pathogen endogenously activates the RAR pathway in Mθs to modulate myeloid programs and, also, its own replication. Considering that RXR also dimerizes with other NRs, in the presence of Vitamin A some sort of cooperation may exist, for instance, with LXRs or PPARs (73). In contrast, Trasino et al. observed that in experimental Vitamin A deficiency, the expression of RARE genes in the lungs of tuberculosis-infected mice is diminished along with an increase in the expression of proinflammatory genes, such as TNF and IL-1β and minimal differences in bacterial growth (74).

#### 3.1.3 PPAR

*Mycobacterium tuberculosis*-infected foamy Mθs (FMs) represent the hallmark of TB lesions. FM refers to Mθs that phagocytose excess lipids and have bubble-like lipid bodies in their cytoplasm, taking on a foamy shape. Not only the morphology of FMs but also their function, with a reduced capacity for both phagocytosis and antimicrobial activity, are altered during infection. Besides, they also provide a nutrient source for *Mycobacterium tuberculosis*, facilitating the long-term survival of the mycobacteria. FMs are mainly located in the granuloma environment, and their death causes the release of their lipid droplets leading to caseum formation and the spread of infection. Regarding the events involved in tuberculous foam cell formation, Ye et al. demonstrated that PPAR receptors are crucial (75).

Several lipid-sensing NRs including PPARγ, PPARδ, but also LXRs, and their heterodimerization partners RXRα and β are expressed in Mθs, with their expression levels and ligand-dependent activities being tightly regulated by various microenvironmental signals. During *Mycobacterium tuberculosis* infection, PPARγ and its target genes are induced, leading to increased lipid droplet accumulation, which activates LXRs, stimulating cholesterol efflux. This receptor regulates CD36 expression and oxidized LDL uptake. It is thought that PPARγ links oxidized LDL uptake to efflux, enhancing lipid flow from Mθs. Inhibition of PPARγ, either by direct antagonists or by stimulation of upstream regulators such as VDR, changes the cumulative lipid content of infected Mθs and increases bacterial intracellular growth (75, 76).

A study from our group shows that PBMCs from newly diagnosed pulmonary TB patients exhibit increased PPARγ transcript levels compared to healthy controls. This increase correlates with lung involvement, pro-inflammatory plasma mediators, and cortisol levels. These findings indicate an attempt to manage the significant inflammatory response at diagnosis addressed to reduce tissue damage and restore homeostasis. Additionally, the concurrent rise of plasma cortisol levels and PPARγ transcripts with disease severity may further reflect the degree of immuno-endocrine-metabolic imbalance (77).

#### 3.1.4 LXR

The metabolism of intracellular pathogens like *Mycobacterium tuberculosis* and their host cells is closely linked. In the case of these mycobacterias, lipids act as essential mediators, by providing nutrient sources for the pathogen or modulating the host immune response. Host cholesterol plays a critical role in the mycobacteria persistent infection, indicating that modulating cholesterol metabolism might constitute a potential strategy for this infection control. A crucial factor in TB is the accumulation of *Mycobacterium tuberculosis*-infected FMs that contain large lipid bodies, impacting host inflammation and bacterial clearance (76). Oxysterols and their LXRs significantly regulate the host immune response to the mycobacteria. Ahsan and colleagues demonstrated that *Mycobacterium tuberculosis* infection induces IL-36 production through the TLR/MyD88 pathway, with IL-1β and IL-18 further promoting IL-36γ synthesis. This cytokine subsequently stimulates the production of LXR ligands, which further elicit the synthesis of antimicrobial peptides like cathelicidin and defensins, enhancing the control of mycobacterial infections (78–80). Interestingly, *Mycobacterium tuberculosis* has developed mechanisms to inhibit oxysterols, known to play a vital role in innate defensive response against this pathogen. A recent study shows that *Mycobacterium tuberculosis* produces enzymes that metabolize host oxysterols, suggesting a potential immune evasion strategy. Since these oxysterols have immunomodulatory and antimycobacterial effects, it is hypothesized that *Mycobacterium tuberculosis* targets them to evade the immune response and persist in Mθs. Additionally, *Mycobacterium tuberculosis* may counteract the oxysterol response by producing antagonists to oxysterol receptors (78).

In addition, was shown that LXR deficiency in mice increases susceptibility to *Mycobacterium tuberculosis*. Mice lacking both LXR α and β isoforms showed diminished clearance of mycobacteria from the lungs, spleen, and liver over several weeks of infection. This impairment was associated with fewer lung-infiltrating neutrophils in LXRα-deficient mice and a decrease in neutrophil-attracting chemokines (81). Also, polymorphisms in this gene are associated with active TB in human patients, indicating that cholesterol homeostasis may underlie the interindividual variation in TB susceptibility (76, 82).

#### 3.1.5 VDR

Some studies suggest that calcitriol, the active form of Vitamin D, enhances the antimicrobial effects of Mθs and monocytes, key effector cells against pathogens like *Mycobacterium tuberculosis*. In addition to boosting the chemotaxis and phagocytic functions of innate immune cells, the calcitriol, VDR, and RXR complex directly stimulates the transcription of antimicrobial peptides, including defensin β2 and cathelicidin. Cathelicidin plays a role in activating the immune response and regulating cytokine and chemokine release (63). Furthermore, experimental studies on BALB/c mice indicated that VD3 is linked to reduced production of inflammatory cytokines in the lungs. Vitamin D supplementation also lowered proinflammatory cytokine levels and the number of Mθs and neutrophils in bronchoalveolar lavage (83), while decreasing cholesterol accumulation in Mθs (84).

Vitamin D is under investigation for its potential to prevent and adjunctively treat TB. While some studies indicate that Vitamin D supplementation may improve clinical outcomes in TB patients by enhancing antimicrobial immune response and reducing inflammation, such evidence remains inconsistent. Additionally, some research suggests a limited efficacy of vitamin D in TB treatment and raises concerns over potential side effects and interactions with other medications (85). In addition, some studies also showed that VDR gene polymorphisms are associated to enhanced or diminished risk to develop TB, depending on the ethnicities of studied populations (44, 86). Therefore, future investigations designed to analyze the levels of Vitamin D and VDR polymorphisms in patients with different severity of disease, may help to understand the exact role of VDR in TB physiopathology and improve treatments.

### 3.2 Chagas disease

#### 3.2.1 GR

Studies analyzing neuro-endocrine and metabolic responses during the early phase of human CD are scarce. Current understanding largely derives from animal models of *T. cruzi* infection, showing that cytokines released by the immune system significantly impact the HPA axis and the disease progression. Acute *T. cruzi* infection induces the release of IL-1β, IL-6, TNF-α, and IFN-γ, leading to a marked increase in corticosterone blood levels. Comparative studies of susceptible C57BL/6 and resistant BALB/c male mice suggest that disease susceptibility is influenced more by the timing and degree of HPA axis activation than by differences in parasitemia (16, 87–89).

In contrast, data on immune-neuroendocrine and metabolic changes in the chronic phase primarily derive from studies in human CD. Our laboratory has demonstrated that pro-inflammatory cytokines are produced during this stage, particularly in symptomatic cases. This inflammatory environment may disrupt endocrine mechanisms, further disturbing the disease course and leading to HPA axis abnormalities. For example, patients with cardiac involvement, the most common dysfunction, exhibit a high cortisol/DHEAs ratio due to a significant reduction in DHEAs while GC levels remain nearly intact or slightly diminished. GR involvement in this pathology, GR-α expression in PBMC of chagasic patients with cardiopathy shows no changes (88, 89).

#### 3.2.2 PPAR

Adipose tissue is a key inflammatory site during the progression of CD and acts as a reservoir for parasites. Infection of cultured adipocytes with the Tulahuen strain of *T. cruzi* leads to increased expression of proinflammatory mediators as well as a marked decrease in PPARγ transcripts (90, 91). Moreover, experimental *T. cruzi* infections may result in body weight loss and significant adipose tissue depletion, likely due to immune–endocrine disruptions and heightened energy expenditure. In this context, PPARγ is necessary for maintaining the mature adipocyte phenotype; its down-regulation likely causes a reduction in metabolic enzymes and adipokines, resulting in an inflammatory phenotype characterized by the secretion of TNFα and IL-6 (91). Furthermore, it was shown that some PPARα and PPARγ ligands can influence the M1/M2 polarization of Mθs infected with *Trypanosoma cruzi*, suggesting a potential pharmacological use of agonists to favor the response against parasite (92).

Cardiac tissue is an important target of *T. cruzi* infection, making it crucial to control the inflammatory response in the heart to prevent fibrosis and remodeling, which can lead to dilated cardiomyopathy and myocardial dysfunction. Previous studies have indicated that PPARγ agonists exert protective, anti-inflammatory effects in *in vitro Trypanosoma cruzi*-infected cardiomyocytes (93, 94). Silencing of PPARγ in cardiomyocytes by small interfering RNA transfection weakens the effects of PPARγ agonist 15-deoxyΔ12,14-prostaglandin J2 (15dPGJ2) on the modulation of pro-inflammatory enzymes. Therefore, enhancing PPARγ expression and activation through endogenous or synthetic agonists may alleviate inflammation, benefiting cardiac and adipose tissues. In this regard, González et al. observed that agonists such as 15dPGJ2 and the synthetic agonist rosiglitazone failed to counteract the expression of pro-inflammatory cytokines in *Trypanosoma cruzi*-infected animals, although the cardiac inflammatory infiltrate was markedly reduced (91).

#### 3.2.3 VDR

Some studies suggest Vitamin D as a potential risk factor for cardiovascular disease. Low serum levels of Vitamin D are often observed in patients with terminal heart failure, highlighting its immunomodulatory role (95). In CD, lower Vitamin D levels would be associated with the cardiac form. A recent *in vitro* study by Dos Santos Oliveira et al. found that serum VD3 levels were lower in patients with CCC compared to those from the indeterminate form. Furthermore, treating PBMCs from both groups of patients with Vitamin D reduced IL-10 production in the cells from cardiac patients, while IL‐2 and IL‐4 levels showed no significant changes post-treatment (86).

An analysis of single-nucleotide polymorphisms of the VDR gene revealed that one of the evaluated alleles was associated with CCC and was involved in the susceptibility to infection by *Trypanosoma cruzi*. Data from such a report suggest that a particular variation within the VDR gene may affect the immune response against the parasite, increasing the probability of cardiac complications in infected individuals. The study of VDR gene polymorphisms in other infectious diseases like TB and leprosy, showed that the same allele conferred susceptibility to CD (96), but their precise role in the pathophysiology of CCC still deserve investigation.

## 4 Discussion

Worldwide, substantial funds are invested in treating infectious diseases. With the sharp rise in pathogens resistant to current therapies (antibiotics, antivirals, antiparasitic), it is essential to explore novel host-directed therapeutics for combating these diseases. While drugs targeting NRs are commonly used for diabetes, atherosclerosis, and autoimmune diseases, their potential in treating infections is gaining recognition. Thus, targeting NRs may open a new largely uncharted avenue for infectious disease treatment. The fact that certain NRs mediate either resistance or susceptibility to infection further underscores the need for both basic science and translational research to unveil NRs prone to being targeted according to the specific infectious disease. In this sense, is essential to identify the upstream steps of NRs activation. Furthermore, another issue worth analyzing is the interactions between NRs and the signaling pathways of pattern recognition receptors such as TLRs, NLRs, and others.

Wager et al, highlight that therapies targeting NRs must consider their diverse roles in regulating physiological processes i.e., metabolism, and reproduction, as well as their involvement in diseases such as autoimmunity, obesity, and atherosclerosis. It is worth noting that when using agonists or antagonists, it is crucial to avoid compromising the body’s natural antimicrobial response (97). In this regard, synthetic GCs, like dexamethasone and prednisolone, which offer enhanced potency and longer half-lives, remain essential for treating inflammatory and autoimmune disorders, and certain cancers. However, their prolonged use leads to significant effects and therapy resistance, and even the reactivation of infectious diseases (98), highlighting the necessity for alternative strategies targeting GR (15).

**Figure 1:**
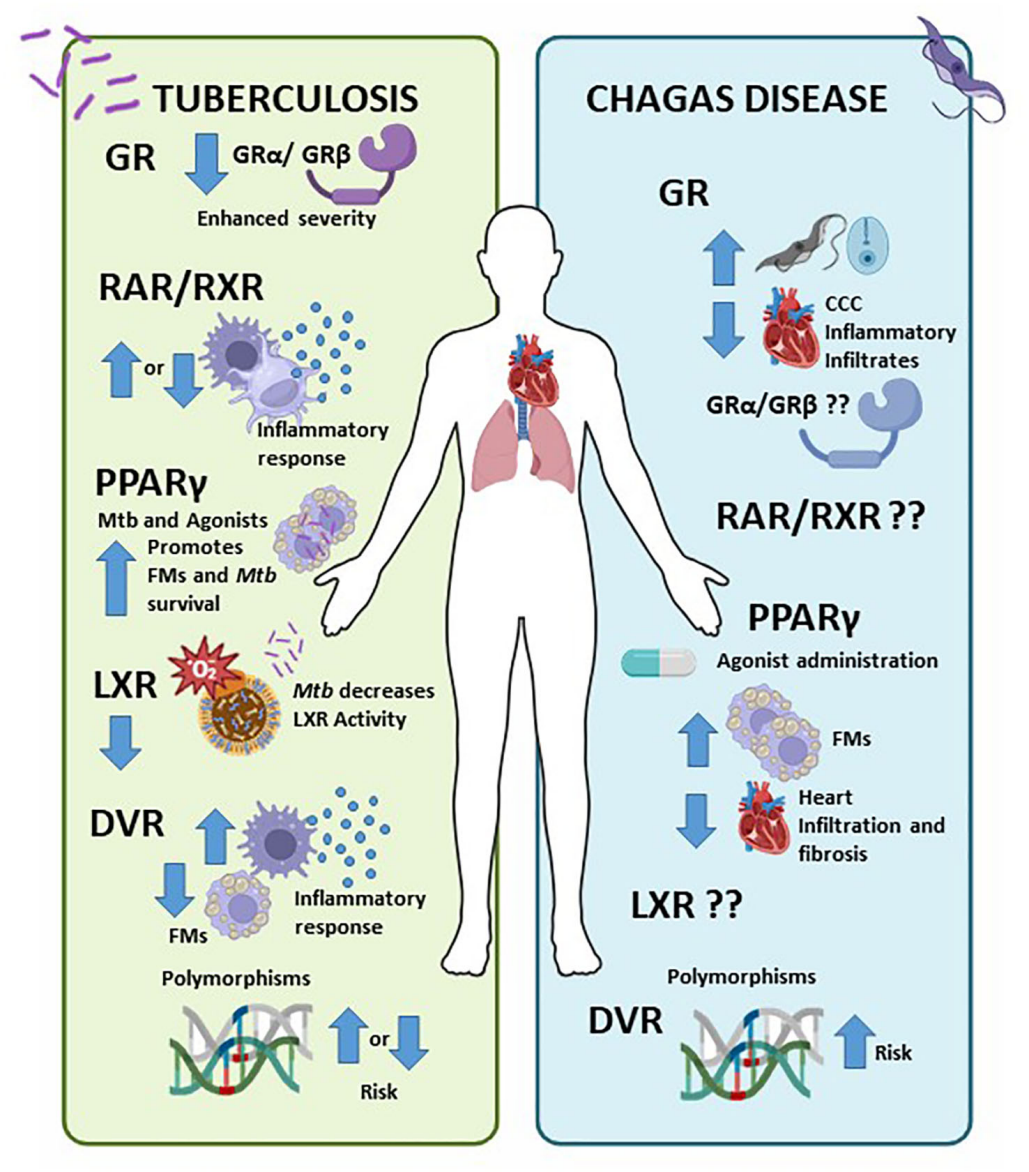
Nuclear Receptors (NRs) involvement in Tuberculosis (TB) and Chagas disease (CD). Left panel) NRs influence the immune response to TB, with higher GRβ and lower GRα/GRβ associated with worse disease severity (70, 104). Research on RAR/RXR yields mixed results; some studies indicate that vitamin A deficiency increases infection risk (71, 72), while others suggest it decreases it (73). PPARs promote anti-inflammatory responses and foamy macrophage formation (75), whereas LXR inhibition benefits *Mycobacterium tuberculosis (* 79). Although Vitamin D has pro-inflammatory effects, certain VDR polymorphisms may facilitate mycobacterial infection, while others reduce the risk of active TB (85). Right panel) In CD, cortisol elevation raises parasitemia, while decreased cortisol favours inflammatory infiltration in CCC (17, 87). PPAR agonists promote FM formation and reduce cell infiltration in cardiomyocytes and heart fibrosis (91, 93). Additionally, certain VDR polymorphisms heighten the risk of CD (96, 105). The involvement of RAR/RXR and LXR in chronic CD is still unknown.

The life cycle of *Mycobacterium tuberculosis* hinges on its interaction with the immune system: it evades innate immunity, survives adaptive immunity without causing symptoms, and triggers a strong inflammatory response that leads to significant tissue damage to facilitate transmission (99). Considering the epidemiology and treatment challenges of TB, host-directed therapy alongside traditional antibiotic treatment is a promising new approach. Numerous studies have explored the neuro-immuno-endocrine response in TB, primarily focusing on the role of several NRs in the inflammatory response (100–103) (**Figure 1**, left panel). Our group found that elevated GRβ expression levels and lower GRα/GRβ ratios are associated with increased disease severity (104). Additionally, research on RAR/RXR shows mixed results, with some studies indicating that vitamin A deficiency raises infection risk (71, 72), while others suggest the opposite (73). PPARs promote anti-inflammatory responses and the formation of foamy macrophage (75), but LXR inhibition benefits *Mycobacterium tuberculosis (* 79). While Vitamin D exhibits pro-inflammatory effects, certain VDR polymorphisms may elevate the risk of mycobacterial infection, while others may reduce the risk of active TB (85).

As comment before, CD is an important endemic parasitic disease in the Americas and now represented a significant public health challenge in the word. Current treatment options are limited to two orally administered antiparasitic drugs, nifurtimox, and benznidazole, which are only effective during the acute phase, targeting amastigotes, and with a cure rate of 50–80%. If left untreated, chronic infection can lead to sudden death from heart arrhythmia, heart failure, or stroke. There is currently no effective treatments for the chronic phases of CD, nor a preventive or therapeutic vaccine (106). The role of different NRs in *T. cruzi* infection is largely unexplored and merits further investigation (**Figure 1**, right panel). In CD, elevated cortisol increases parasitemia, whereas reduced cortisol promotes inflammatory infiltration in CCC (17, 87). PPAR agonists enhances FM formation and decrease cell infiltration in cardiomyocytes, reducing heart fibrosis (91, 93). Certain VDR polymorphisms also elevate CD risk (96, 105). The role of RAR/RXR and LXR in chronic CD it remains unclear.

Host-directed therapies present a promising opportunity to develop new treatment strategies for infectious diseases. TB and CD pose significant public health challenges, necessitating approaches that target both the pathogen and the host. Novel therapies involving NRs modulation can regulate the immune response to reduce pathogen replication and enhance patient outcomes.
